# Characterization of a Geopolymer Foam by X-ray Tomography

**DOI:** 10.3389/fchem.2021.754355

**Published:** 2021-12-24

**Authors:** Svetlana Petlitckaia, Jérôme Vincente, Arnaud Poulesquen

**Affiliations:** ^1^ CEA, DES, ISEC, DE2D, SEAD, LCBC, Université of Montpellier, Marcoule, France; ^2^ Aix Marseille Univ, CNRS, IUSTI, Marseille, France

**Keywords:** geopolymer foam, x-ray tomography, iMorph software, permeability, formulation

## Abstract

Metakaolin based geopolymer foams were synthesized at room temperature by direct foaming using hydrogen peroxide (H_2_O_2_) as a blowing agent and two types of surfactants such as AER5 and CTAB allowing to tune the connection between two adjacent cells. In the field of decontamination process of liquid wastes, the knowledge of the topology of the generated macroporous network is a primary of interest. Due to the complex structure of porous material, 2D conventional techniques as optical or scanning electron microscopy are often not able to provide all the necessary informations. The 3D networks were therefore characterized by X-ray tomography to determine the morphological structure parameters that is useful to manufacture geopolymer material for filtration applications. The porosity, the pore size distribution and constriction between adjacent cells, as well as the connection rates between pores were analyzed by the iMorph program. The results show that the total porosity increases from 26 to 74% when the initial concentration of H_2_O_2_ increases, which is in complete agreement with the tomography results. Materials synthetized from CTAB surfactant are poorly connected whereas those generated from AER5 surfactant have a higher mean cell size (at equivalent initial H_2_O_2_ concentration) and are fully connected, which will facilitate the transport of fluid through the material. These features have a strong impact on the value of permeability coefficients of the geopolymer foams. Indeed, permeabilities calculated from a Pore Network Modeling (PNM) approach or Kozeny-Carman equation, are ranged in between 10^−14^ to 10^−10^ m^2^ depending on the cell connectivity, the throat size and the total porosity.

## 1 Introduction

Geopolymers (Davidovits, 1991) are amorphous three-dimensional inorganic materials that can be produced by alkaline activation of aluminosilicate sources at ambient temperature (Duxson et al., 2007)**.** This material has a structure containing tetrahedrally coordinated aluminium and silicon atoms. The deficit of the charge balance of tetrahedral Al is compensated by the presence of alkali such Na^+^ ou K^+^ (Barbosa and MacKenzie, 2003; O’Connor et al., 2010; Steins et al., 2012; Skorina, 2014; Benavent et al., 2016; Luukkonen et al., 2016). Consequently, geopolymer can be viewed as an amorphous analogue of a zeolite capable of cations exchange (Bortnovsky et al., 2008; Sazama et al., 2011). Geopolymer properties are strongly dependent on the raw materials, synthesis parameters and curing condition (Luukkonen et al., 2018) and the different research area show that final properties can be tailored by the chemical composition of the mixture.

The application fields of geopolymer are based on the final properties and the structure or shape of the synthetized material. Indeed, geopolymers may be processed as monolith, granule, powder, foam or by 3D printing that may be useful according to the targeted application (Franchin et al., 2017; Novais et al., 2018; Petlitckaia and Poulesquen, 2019; Medri et al., 2020). If well formulated, alkali activated materials have a high compressive strength, good chemical and thermal resistance and good aging properties and durability, which are beneficial for a number of industrial applications (Sazama et al., 2011; Novais et al., 2020)**.** Geopolymers show promising potential to be used in the civil engineering (Kamseu et al., 2012; Yan et al., 2019; Ricciotti et al., 2020), chemical industries (Cheng et al., 2012) but also in nuclear field for many applications such as the stabilisation of liquid nuclear oil waste (Cantarel et al., 2018), the encapsulation of Mg-Zr cladding (Rooses et al., 2013) or the decontamination of radioactive effluents using lightweight geopolymer as a solid support (Petlitckaia et al., 2020).

Many works address the synthesis and use of lightweight (macroporous) geopolymer. The foams can be synthesized by different technique such as emulsion templating (Medpelli et al., 2014; Barneoud-Chapelier et al., 2020), ice-templating (Papa et al., 2016) and direct foaming (Prud’homme et al., 2011; Landi et al., 2013; Strozi Cilla et al., 2014; Petlitckaia and Poulesquen, 2019). The former is the most used technique to produce geopolymer foam. This is achieved by adding different type of blowing agent (H_2_O_2_, metal aluminum) generating gas which in turn create the macroporosity. The bubbles of gas O_2_ are then trapped into the geopolymer matrix. Adding hydrogen peroxide allows to produce a more homogeneous foam (Bai and Colombo, 2017; Petlitckaia and Poulesquen, 2019). The amount of H_2_O_2_ incorporated affects the mechanical and physical properties of geopolymers. The use of ionic, non-ionic or amphiphilic surfactants in the geopolymer paste is helpful to stabilize the gas bubbles but their uses modify the workability and the topology of the final materials (open or closed porosity).

The characterization of the porous network of the lightweight geopolymer such as the total pore volume, pore size distribution from nano to macro scale, tortuosity, connectivity and pore wall thickness is of primary of importance. Many techniques can be used and combined to fully characterize the porous network. Gas adsoprtion or mercury intrusion porosimetry allow to compute the pore volume, the specific surface area and the pore size distribution (at different space scale) but these methods can give distorted results due the breaking of the inner pore’s walls (Bogas et al., 2012; Korat et al., 2013) especially for MIP or simply due to the preparation method of the samples before characterization. Moreover, these methods are considered as invasive or destructive one. Microscopy is also very useful to observe the samples from nano to microscale by giving some informations on the precipitates, often combined with chemical analysis, and the shape and topology of the porous network. Unfortunatly, it is a 2D technique that skew the pore size analysis and the sample preparation may also modify the analysed surface. Consequently, those conventional techniques are not capable of providing all necessary important information such as total porosity, size of inner cavities, orientation of the cells, connectivity between the cells.

This paper aims to fully characterize the structure of the macroporous network of a geopolymer foam by using X-ray tomography (Bourret et al., 2014). It is a non destructive technique that allow to observe by reconstruction, the 3D network and compute many quantities as total porosity, pore size distribution, connectivity etc. The influence of formulation parameters such as the initial concentration of H_2_O_2_ and the nature of the surfactant was assessed and compared with experimental data. We report a good correlation on the total porosity between the experiments and the 3D data for all the tested samples. From the topology of the macroporous network characterized by tomography, we compute the permeability that clearly increases when the initial concentration of H_2_O_2_ is high (due to an increase of the pore and throat size) combined with the use of anionic surfactant (AER5). The results is in a good agreement with the empirical Kozeny-Carman equation.

## 2 Materials and Methods

### 2.1 Material

The geopolymer foams were synthesized using commercial sodium silicate solution (Woellner, Betol 39T: 27.8% SiO_2_, 8.3% Na_2_O and 63.9% H_2_O), sodium hydroxide (Sigma Aldrich, 99%) and alumino-silicate source (metakaolin, ARGICAL-M 1000, Imerys) (Table 1). Hydrogen peroxide (50% w/w, density = 1.19 g cm^−3^, M = 34.015 g mol^−1^, Sigma-Aldrich) was used as chemical foaming agent. A commercial air entraining Sika AER5 which has similarity with SDS (density, 1.035 g cm^−3^; pH, 11) and cationic surfactant CTAB (cetyltrimethyl ammonium bromide CH_3_(CH_2_)15N(Br)(CH_3_)_3_, Sigma Aldrich) were used as surfactants (Table 2).

**TABLE 1:** Chemical composition of raw materials.

Materials	Weight %								
	SiO_2_	Al_2_O	CaO	Fe_2_O_3_	TiO_2_	K_2_O	Na_2_O	MgO	H_2_O
Metakaolin Argical M1000	54.4	38.4	0.10	1.27	1.6	0.62	0.2	0.2
Betol^®^39T Woellner	27.8						8.3		63.9

**TABLE 2:** Characteristics of blowing agent and surfactants used for the synthesis of geopolymer foams.

Materials	Production	Type		Density, kg/m^3^	pH	Composition
H_2_O_2,_ 50% w/w	Sigma Aldrich	Blowing agent	liquid	1,190	11 ± 1	Unknown but mainly anionic, 1–2.5% tall oil selt 1–2.5% K_2_CO_3_
SIKA^®^ AER5	Sika	Surfactant	liquid	1,035	11 ± 1	
CTAB	Sigma Aldrich	Surfactant	powder			1–2.5% tall oil selt 1–2.5% K_2_CO_3_

### 2.2 Geopolymer Foam Preparation

**FIGURE 1:**
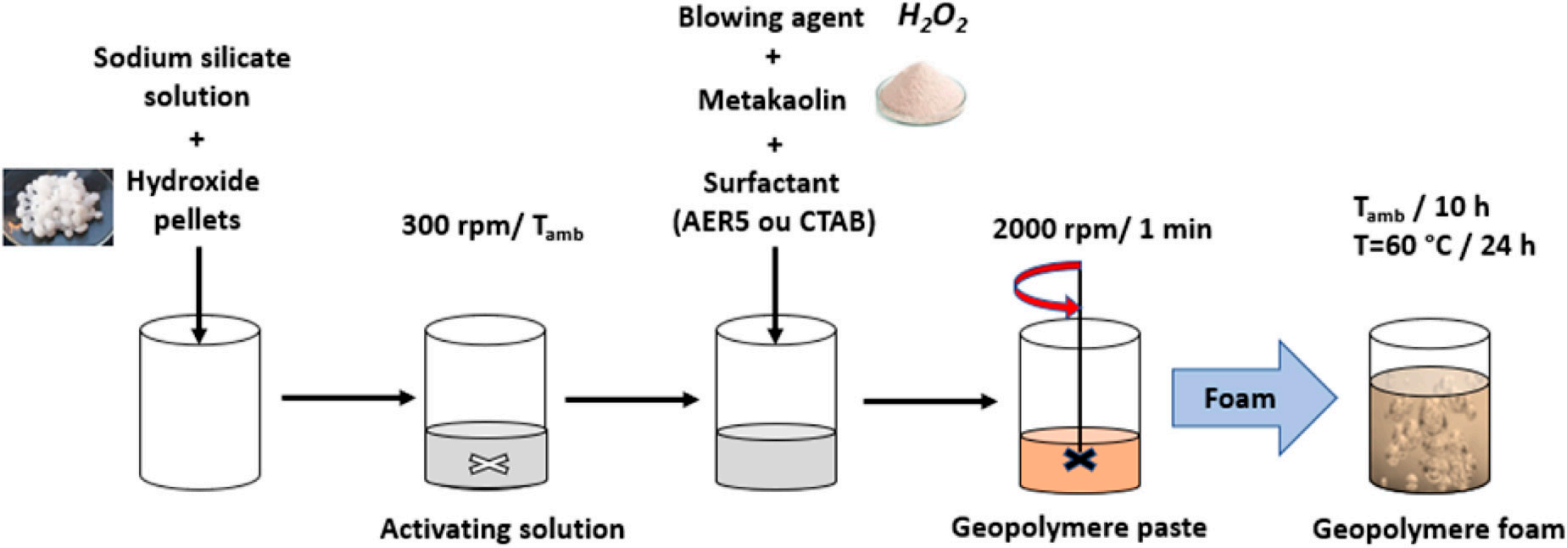
Geopolymer foam synthesis protocol.

Geopolymer foams were synthesized with a molar composition SiO_2_/Al_2_O_3_/Na_2_O/H_2_O = 3.6/1/1/12. Figure 1 present geopolymer foams synthesis protocol. Sodium hydroxide pellets were totally dissolved in aqueous sodium silicate by magnetic stirring at 300 rpm (activating solution) until the pellets were totally dissolved. The dissolution reaction being exothermic, stirring was prolonged until the solution returned to room temperature. Samples were prepared by mixing the activating solution, surfactant, metakaolin and foarming agent for 1 min under mechanical stirring at 2000 rpm directly mixed into open cylindrical polystyrene molds. The surfactants AER5 and CTAB were added to the activating solution before adding metakaolin. . Hydrogen peroxide was added at different concentration R = 0.25–1.25% (R being the ratio between the initial volume of H_2_O_2_ [V_H2O_], and initial volume of geopolymer paste [V_0_]). The surfactants AER5 and CTAB were used to optimize the homogeneity (stabilize the gas bubbles) and mecanical resistance of the resulting geopolymer foam. Samples were casted in closed molds ans stored at room temperature for 10 h to promote polycondensation and then placed in an oven at 60°C for 24 h to consolidate the stucture. From previous results (Petlitckaia and Poulesquen, 2019) on this kind of foams, we found that the level of volume expansion (40–475%), the volume fraction of generated gas (28–83%) and the pore size (150–3,000 µm) are all controlled by the initial concentration of H_2_O_2_ (R = 0.25–2.5%) regardless of the surfactant used. However, the nature of the surfactant had a drastic effect on the rheological properties of the two pastes, leading to differences in the morphology and topology of the macroporous networks in the foams. The CTAB based geopolymer consists of more densely packed closed faceted pores, whereas the AER5 based foam consists of a 3D network of interconnected pores.

### 2.3 Characterization Technique

#### 2.3.1 X-Ray Microtomography Experiment

**FIGURE 2:**
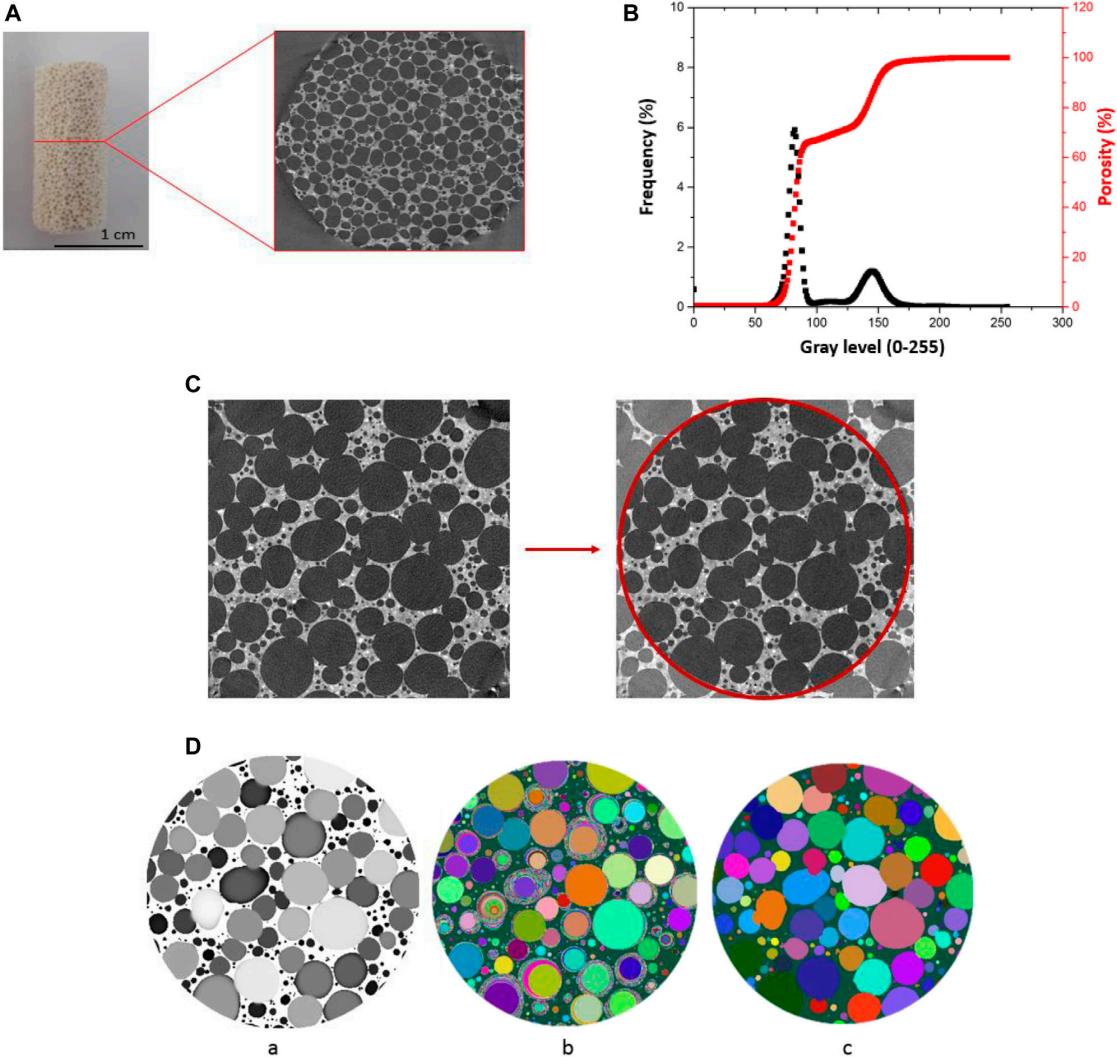
The different steps of tomography measurement and pre-processing for data analysis: **(A)** the sample of geopolymer foam and raw data of x-ray tomography, **(B)** histogram of gray level for analyzed sample, cumulated grey level distribution (%) gives porosity obtained depending on the grey value chosen threshold, **(C)** choice of region of interest (ROI), **(D)** example of morphological analysis performed on a measurement sample where (a)—aperture diameter map, (b) maximal included ball map–and (c)- segmented cells.

The three-dimensional method of X-ray tomography was chosen to determine the morphology of pores and the topology of the poral phase. The acquisition of the three-dimensional images was performed with an Easy TomXL ULTRA 150 tomography at the Mechanical and Engineering Institute–IMI of Marseille. The samples are prepared upstream and positioned inside the conical beam constituted by the X-rays emitted from their emission focus towards the detector. To obtain the smallest voxel size available with the tomograph, the sample should be positioned as close as possible to the X-ray emission point while remaining entirely within the cone of the beam. The samples were cut into cylinders 7 mm in diameter and 20 mm in height (Figure 2A) to obtain voxel size between 5 and 10 µm. The detector is positioned at the greatest possible distance to ensure enough contrast and obtain suitable voxel size. The X-rays passing through the sample are attenuated by absorption and impregnate the detector to form an X-ray projection. These projections are recorded for a complete rotation of the specimen from 0 to 360°. The greyscale X-ray image obtained at the detector is then recorded. Based on all these projections, a Back-Projection algorithm using Xact software is used to provide volume density information (3D reconstruction) where the grey levels of the reconstructed image reflect a local density. We can then access the differences in composition, as well as the presence of heterogeneities (pores, inclusions, *etc*.) within the sample.

#### 2.3.2 3D Image Analysis Method

**FIGURE 3:**
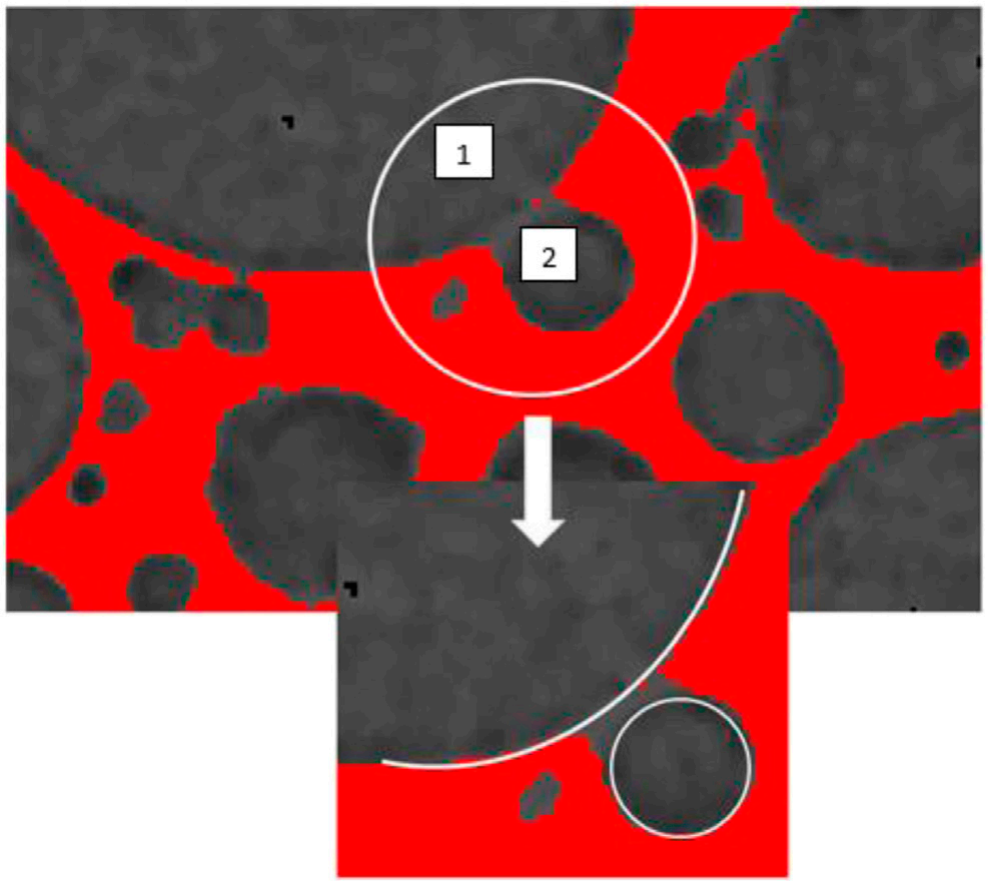
Setting of threshold performed on a foam synthetize with CTAB surfactant and R = 1% v/v (where volume of geopolymer paste v_0_ = 30 ml and volume of H_2_O_2_ v_1_ = 0.3 ml).

To perform the 3D quantitative analysis the different phases should first be identified. A first image preprocessing method was applied to treat the gray level images based on an initial noise reduction step using median filters with radius r = 2. The binarization process is obtained by selecting a threshold determined from the analysis of the histogram of grey levels composing the image. As we have strong contrast between air and solid matrix it is possible to separate the poral phase (peak between 60 and 100) from the solid (peak between 120–170) (Figure 2B). However, if the resolution is insufficient, we will not be able to detect very thin walls separating pores, conversely if the threshold is too low, more solid voxels will be detected and we can artificially block very narrow passages and limit the size of the percolated network (Figure 3).

After binarization process morphological operators are applied to remove isolated solid voxels, pores cavities are kept.

The next step is the choice of region of interest (ROI) used for the analysis (Figure 2C). To be statistically representative of the sample, the ROI must be large enough to contain a certain number of complete and fully enclosed cells. In this study the selected ROI is a centered cylinder of 635 voxels in diameter and 854voxels in height, correspond to an analyzed volume of Ø6.35 mm × 8.54 mm or Ø3.17 mm × 4.27 mm depending on the used voxel size.

3D quantitative analysis of the poral space is made using iMorph software (Vicente et al., 2006). The different steps of segmentation of cells are demonstrated in Figure 2D. The segmentation uses a watershed markers-based method where markers are obtained from the maximal balls’ identification. This made the determination of both cell shape and orientation possible and gave additional information about the three-dimensional structure.

This segmentation ensures that all cells contain a single maximum ball and are necessary surrounded by constrictions (throats) that connect other cells. The throats that are the passage windows between pores correspond to the topological constrictions of the poral space. Therefore, it is interesting to know their size and morphology. From the previous automatic pore segmentation using watershed, voxels located at the passage window between two adjacent pores constitute a surface called constriction or throats. The diameter of the disc with the same surface is also calculated as well as the diameter of the inscribed disk.

#### 2.3.3 Pore Size Distribution and Characteristic Diameters (Dn)

**FIGURE 4:**
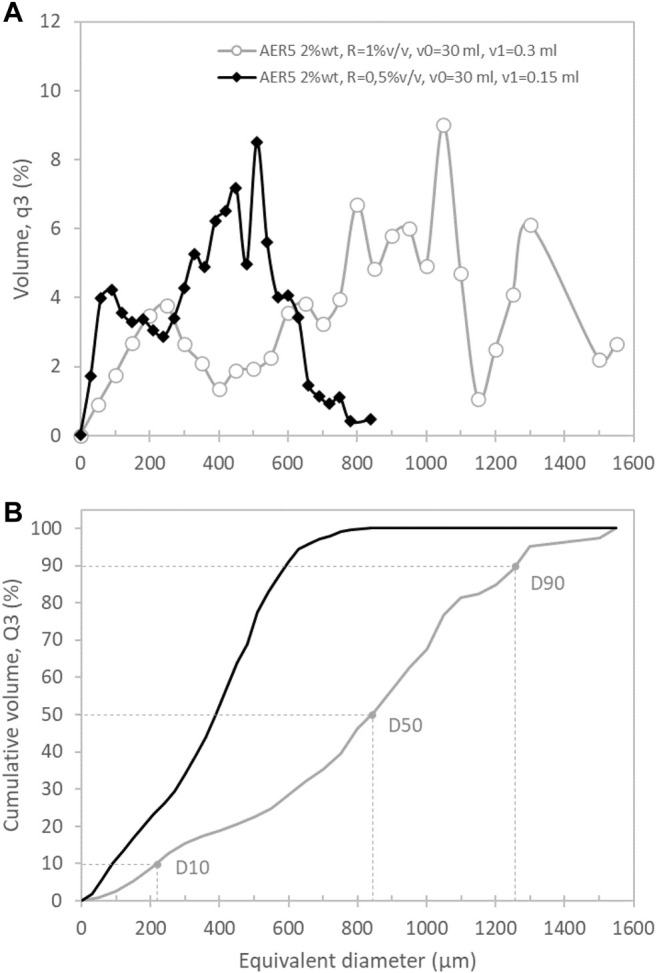
Pore size distributions for the operating conditions AER5 2%wt for S3 {R = 1%v/v, v0 = 30 ml, v1 = 0.3 ml} and S7 {R = 0.5%v/v, v0 = 30 ml, v1 = 0.15 ml}. These distributions are expressed: **(A)** in percentage of volume (q3) and **(B)** in percentage of cumulative volume (Q3). Each bin is 30 µm wide. D 10, D 50, and D 90 are also represented on the cumulative curve of S7.

Based on the 3D pore segmentation, the equivalent pore diameter size distributions were then calculated according to the standard ISO 9276-1 (1998) for particle size analysis (ISO - ISO 9276-1, 1998)**,** and expressed in terms of percentage of volume (q3) and cumulative volume (Q3). For practical applications related to particulate separation or porous material characterization, it can be easier to use the characteristic equivalent diameter *Dn* than the full distributions: such a metrics corresponds to the cell diameter for which n% of the volume distribution has smaller cell sizes and (100-n)% has larger cell sizes. In Figure 4B, we present D10, D50 and D90, which thus correspond to the abscissas obtained for 10, 50 and 90%, respectively, from the cumulative distributions of Figure 4B.

Characteristic equivalent diameter of throats, that are 2D surface objects by construction, can also be determined using the equivalent throat diameter size distributions expressed in terms of percentage of throat surface (q3) and cumulative surface (Q3).

#### 2.3.4 Permeability Computation With Pore Network Approach

The usual conceptual representation of PNM (Pore Network Modeling) divides the void space into nodal pores connected by channels containing throats. The gravity center of constrictions voxels, and of cells voxels constitutes the constrictions nodes and cells nodes of the pore network respectively. To complete this network, we identify the cells in contact with the inlet and outlet faces of the simulation. The center of these pore faces at the inlet or at the outlet constitutes the boundary nodes of the porous network thus obtained. The morphological data of the cells constrictions and cells boundary faces are assigned to their corresponding network nodes. For the simulation we choose as local diameter, the inscribed spheres diameter for cell nodes and the inscribed disc diameters for constrictions and boundary nodes. Absolute permeability calculation is performed directly on the extracted pore/throat network. The principle of permeability calculation has been reported previously by (Laroche and Vizika, 2005). Geometric shapes of the pores are usually quite complex. This makes the computation of the local conductance of the pores complicated. Formulas are found in the literature that express the conductance as a function of the surface traversed by the flow rate and the length of the pore, they are generally formulas in simple geometrical shapes: sphere, cube, conics. A simple approximation of the fluid conductance between center pore *i* and center throat *k* of an is defined by:

$$g_{ik}=\frac{\pi \left(r_{i}^{4}+r_{k}^{4}\right)}{4\mu \left(r_{i}^{4}.r_{k}^{4}\right)l_{ik}}$$

Where *r*_*i*_ and *r*_*k*_ are the hydraulic radius of the pore *i* and throat *k* respectively, *l*_*ik*_ is the distance between pore *i* and throat *k, and* μ is the dynamic viscosity (Pa.s).

To be able to model the flow coming from a pore *i* to the neighboring pore *j* passing through a *k*-throat, we compute an effective local conductance *g*_*ij*_ which is the harmonic mean of the two local conductances *g*_*ik*_ and *g*_*kj*_ *= g*_*jk*_ *.*

$$g_{ij}=\frac{g_{ik}*\;g_{kj}}{g_{ik}+g_{kj}}$$

The calculation of the permeability is done with the pore network method by solving the Darcy equation, Eq. 1.

$$Q=\frac{A*\;K}{\mu }*\frac{\Delta P}{L}$$ (1)

whith:

- Q flow rate (m^3^/s)
- K intrinsec permeability (m^2^)
- A  normal cross section to the flow direction (m^2^)
- $\frac{\text{Δ}P}{L}\;$ hydraulic gradient (Pa/m)
- μ dynamic viscosity (Pa.s)

From every pore *i* we assemble the matrix that correspond to the linear system used to compute the pressure *P*_*i*_ of each pore *i*. Each line *i* of the matrix represents the law of Conservation of Mass in the pore *i*, where we can write:

$$\sum_{j\;neighbors\;pores\;}q_{ij}=0$$

where $q_{ij}$ is the mass flux between pore *i* and *j* can be written $q_{ij}=g_{ij}\left(P_{i}-P_{j}\right)$ with $g_{ij}$ the local conductance of pore *i* to pore *j* and *P*_*i*_ and *P*_*j*_ the pression of pore *i* and *j* respectively.

We use boundary conditions of the Dirichlet type, where the pressures of the nodes at the input and output faces are respectively fixed to *P*_*inlet*_ and *P*_*outlet*_. The system is solved and from the pressures computed at each node we can estimates the total flux Q directly from pores located at the inlet or at the outlet faces, and we deduce the permeability from the Darcy equation (Eq. 1). Knowing the pressure at each pores the total Flux Q is obtained from the summation of all the local flux of pores directly connected to the inlet. Flux balance is also verified as the outlet flux should be equal to the inlet flux. Pore size distribution and intrinsic permeabilities computed by pore network modeling are reported on Table 5.

## 3 Results

### 3.1 Influence of the Nature of the Surfactant

**FIGURE 5:**
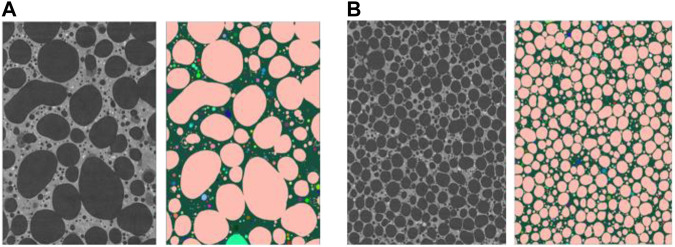
Example of morphological analysis perfomed on a measurement of samples based on based on AER 5 **(A)** and CTAB **(B)** surfactants with the same quantity of blowing agent H_2_O_2_ (R = 1%, volume of geopolymer paste v_0_ = 30 ml and volume of H_2_O_2_ v_1_ = 0.3 ml) (pink color shows percolated clusters and other colors are isolated clusters, vertical cut of cylindrical Roi Ø635 x 854 voxels; S3 **(A)** Ø6.35 mm × 8.54 mm (10 µm voxel size) and S10 **(B)** Ø3.17 mm × 4.27 mm (5 µm voxel size).

From the images obtained by X-ray tomography, the porous structure of monolithic geopolymer foams based on two types of surfactants AER5 and CTAB are compared in Figure 5 **,** for the 30 ml of geopolymer paste and 0.3 ml of H_2_O_2_ (R = 1% v/v). Both samples present the same volume fraction of gas (S3 and S10 Table 3) but we clearly show that the geopolymer foam based on AER5 surfactant contain larger macropores in lower number as compared to the CTAB one. The analysis by iMorph of the connectivity between the cells shows that both materials are fully connected (pink color in Figure 5) with a percolated volume of 97.2 and 98% for AER5 and CTAB based materials respectively (Table 4). However, the pore and throat sizes are dramatically lower for the CTAB based geopolymer (Table 5). This result clearly indicates that the nature of surfactant impacts the pore size distribution by shifting this one to lower value when cationic surfactant is used rather than an anionic one. As suggested elsewhere (Petlitckaia and Poulesquen, 2019), the bulk viscosity of the paste is dramatically modified by using a cationic surfactant due to its strong affinity with the clay particle surface modifying the gas/paste interface.

**TABLE 3:** Compositions and main characteristics of synthetized geopolymer foams (apparent density, volume expansion and volume fraction of gas).

Sample	Volume of geopolymer paste, ml	Surfactant	H_2_O_2_, ml	R, % v/v	Drying	Apparent density, g/cm^3^	Volume expansion,%	Volume fraction of gas
S1	10	AER52% wt	0.1	1	at room temperature	0.548	150	0.600
S2	20		0.2	1		0.626	153	0.605
S3	30		0.3	1		0.598	165	0.623
S4	40		0.4	1		0.598	165	0.623
S5	10		0.05	0.5		0.830	80	0.444
S6	20		0.1	0.5		0.883	84	0.457
S7	30		0.15	0.5		0.877	80	0.444
S8	40		0.2	0.5		0.869	80	0.444
S9	30	CTAB 0,05% wt	0.075	0.25	at room temperature and in the oven at 60°C, 24 h	1.217	35	0.259
S10	30	CTAB 0,05% wt	0.3	1		0.550	172	0.632
S11	20	AER52% wt	0.25	1.25	at room temperature	0.381	275	0.733

**TABLE 4:** Porosity and percolated volume of samples obtained from microtomography images analysis.

Sample	S1	S2	S3	S4	S5	S6	S7	S8	S9	S10	S11
voxel size (µm)	10	10	10	10	5	5	5	5	5	5	10
porosity	60	60.5	62.3	62.3	44.4	45.7	44.4	44.4	25.9	63.2	73.3
porosity from tomography	66,6	63,3	66,8	66,1	45,7	43,3	47,1	48,2	20,6	67,3	75,1
st. dev	5,6	2,6	2,6	3,1	3,1	4,2	3,5	3,8	0,8	1,8	5,6
percolated volume (%)	95,0	95,9	97,2	96,6	29,0	np	np	80,1	np	98,0	98,6

**TABLE 5:** Pore and throat size–Computed Permeability with Pore Network modelling (PNM) comparison with Kozeny-Carman model. ^**(a)**^ Equivalent pore diameter size distributions expressed in terms of percentage of pore volume, ^**(b)**^ Equivalent throat diameter size distributions expressed in terms of percentage of throat surface, ^**(c)**^ mean value of inscribing disk diameter distribution (number), (np) for not percolated toward vertical direction.

	PSD (µm) characteristic eq. Diameter ^(a)^				Throats diameter (µm)		Permeability (m^2^)	
	Mean	D10	D50	D90	D10^(b)^	Inscr.disk ^(c)^	Kozeny-carman	PNM
S1	1,307 ± 569	360	1,360	1950	20	26.4 ± 103.4	3.00E-12	1.11E-11
S2	743 ± 311	230	750	1,160	100	70.8 ± 182.2	7.89E-11	6.89E-11
S3	819 ± 372	230	860	1,285	90	63.6 ± 92.6	7.66E-11	1.87E-10
S4	854 ± 439	250	850	1,325	110	64.2 ± 94.4	1.14E-10	1.53E-10
S5	330 ± 153	105	320	510	15	10.1 ± 15	3.54E-13	4.45E-17
S6	323 ± 192	80	310	495	18	19.5 ± 15.4	5.83E-13	(np)
S7	375 ± 178	90	390	590	15	22.3 ± 19.6	3.54E-13	(np)
S8	371 ± 192	95	430	690	15	18.2 ± 13.3	3.54E-13	7.67E-14
S9	84 ± 94	12	45	142	5	11.8 ± 6.5	4.40E-15	(np)
S10	446 ± 124	340	460	525	12	17.1 ± 13.4	1.49E-12	6.29E-14
S11	980 ± 427	270	1,055	1,490	85	25.4 ± 81.5	2,15E-10	1.88E-10

### 3.2 Influence of H_2_O_2_

**FIGURE 6:**
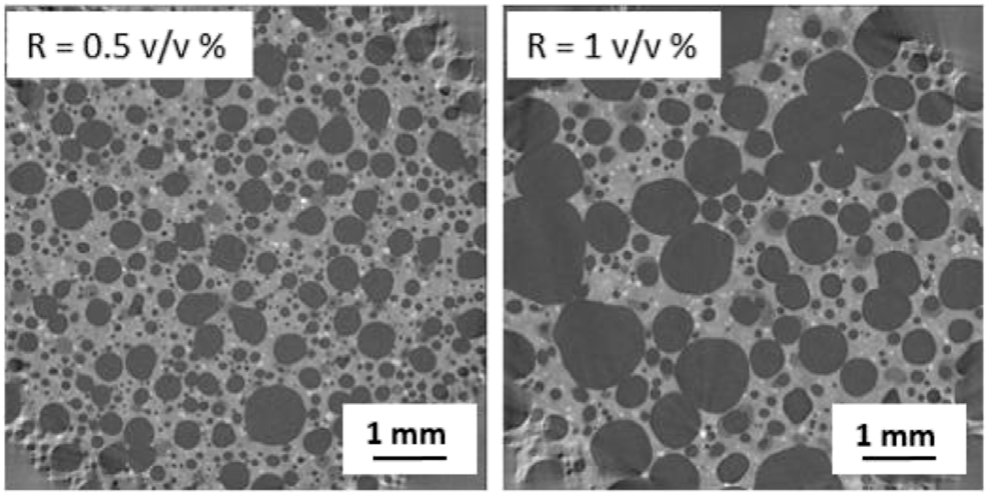
Raw data of X-ray tomography of sample based on AER5 surfactant with R = 0.5 (S7) and R = 1 v/v % (S3) where volume of geopolymer paste v_0_ = 30 ml and volume of H_2_O_2_ v_1_ = 0.15 and 0.30 ml respectively).

As discussed previously, the morphology of the pores depends not only on the chemical nature of the surfactant but also on the amount of H_2_O_2_ added as foaming agent (the R ratio). Increasing the initial concentration of hydrogen peroxide produces a more aerated foam with a higher generated macroporosity (as reported in Table 3, the porosity measured experimentally is 44 and 62% for the R = 0.5 v/v % samples [S5-S8] and R = 1 v/v % samples [S1-S4] respectively) whatever the surfactant used. An increase of the mean pore size is also observed visually as suggested by the raw data of X-ray tomography images (Figure 6 for 0.5 and 1 %v/v that corresponds to S7 and S3 respectively**)**. The full analysis of the 3D acquisition shows a good concordance between the experimental and computed porosity (Table 4) but allows also to quantify the volume pore size distribution (q3) and the cumulative volume (Q3) as a function of the equivalent diameter of cells (Figures 4A,B respectively). For both formulations (S3 and S7), we found a bimodal volume distribution corresponding to two classes of equivalent diameter. These two classes of diameter correspond to the characteristic equivalent diameter D10 and D50 reported on Figures 4B and Figure 9 **.** For instance, 50% of the total pore volume (D50) is represented by equivalent pore diameter lower or equal to 390 and 860 µm for R = 0.5 %v/v or 1 %v/v respectively. Figure 4B shows also that the entire pore volume is represented by pores with equivalent diameter lower than 800 µm for R = 0.5 %v/v whereas it reaches 1,450 µm for R = 1 %v/v. Finally, it is important to mention that the macroporous network is fully percolated for R = 1 %v/v (S3 sample) whereas the S7 sample do not present a percolated volume.

### 3.3 Characterization of the Macroporous for the Selected Formulation (AER5 2% wt, R = 1.25 v/v %)

**FIGURE 7:**
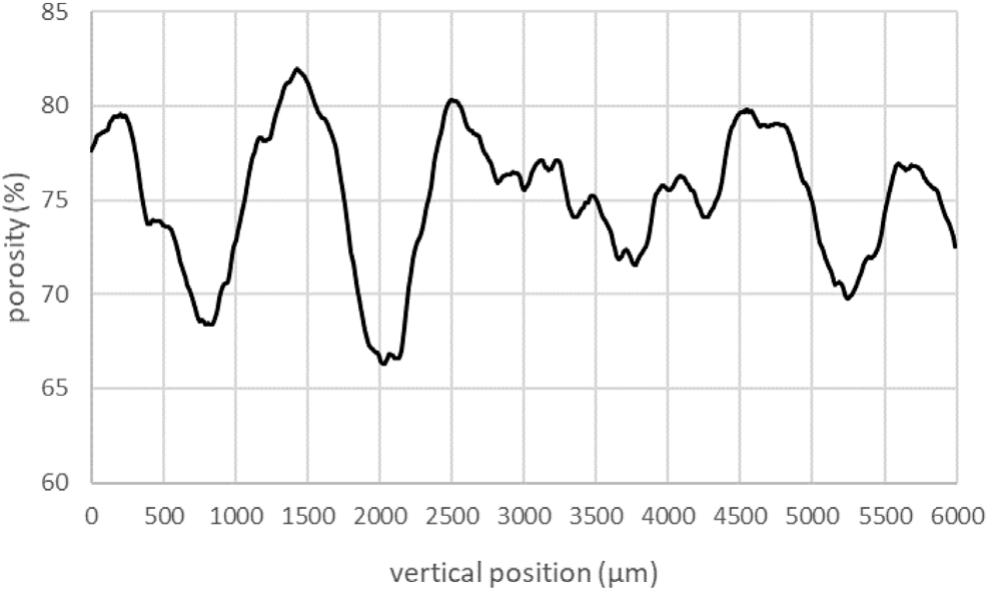
Evolution of porosity along z axis for the geopolymer foam sample S11 produced with AER5 2%wt and R = 0.25 v/v % (where volume of geopolymer paste v_0_ = 20 ml and volume of H_2_O_2_ v_1_ = 0.25 ml).

The formulation with surfactant AER5 2% and R = 1.25 v/v % (S11 sample in table 3) was chosen as the optimal formulation for the decontamination of liquid wastes as already discussed elsewhere (Petlitckaia et al., 2020). The macroporous geopolymer foam with a gas volume fraction of 73%, a density of 0.381 g/ cm^3^ and a compression strength of 0.77 MPa was therefore synthesized for this specific application. Fully characterization by X-ray tomography was done in order to have more specific topological and structural information such as the cell and throat sizes, the connectivity, local porosity where its evolution along the z axis shows an amplitude of about 13% (Figure 7)**.** The mean porosity computed by iMorph is in complete agreement with the experimental value reported in Table 4.

**FIGURE 8:**
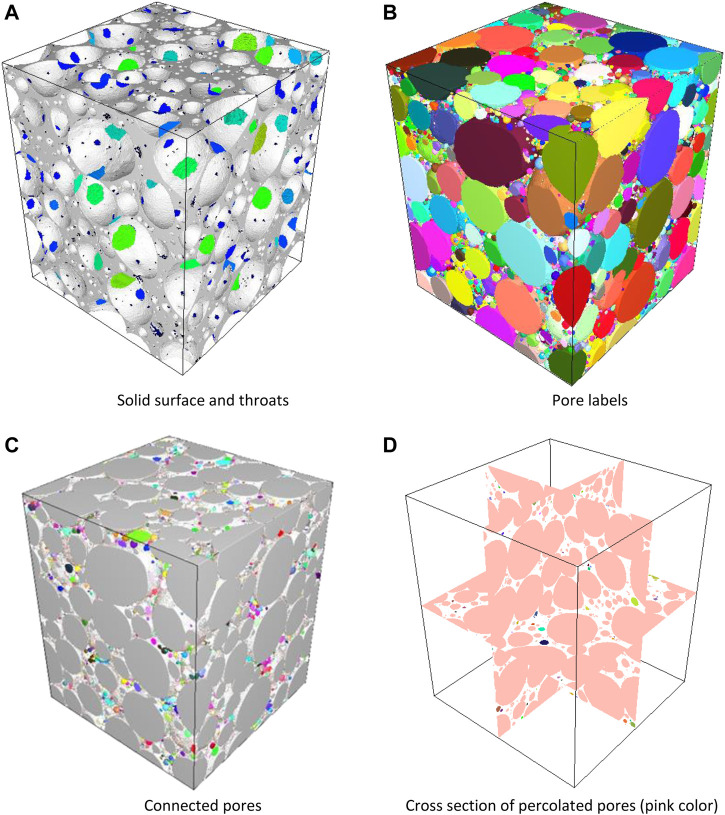
visualization of reconstruction of geopolymer foam S11 produced using AER 5 2 % wt as surfactant for R = 1.25 v/v % (where volume of geopolymer paste v_0_ = 20 ml and volume of H_2_O_2_ v_1_ = 0.25 ml) **(A)** visualization of throat colored according to their respective sizes. **(B)** 3D pores colored according to their cells label. **(C)** percolated pore in grey, colored pores are not connected cavities, and **(D)** cross section of percolated pore (pink color).

**FIGURE 9:**
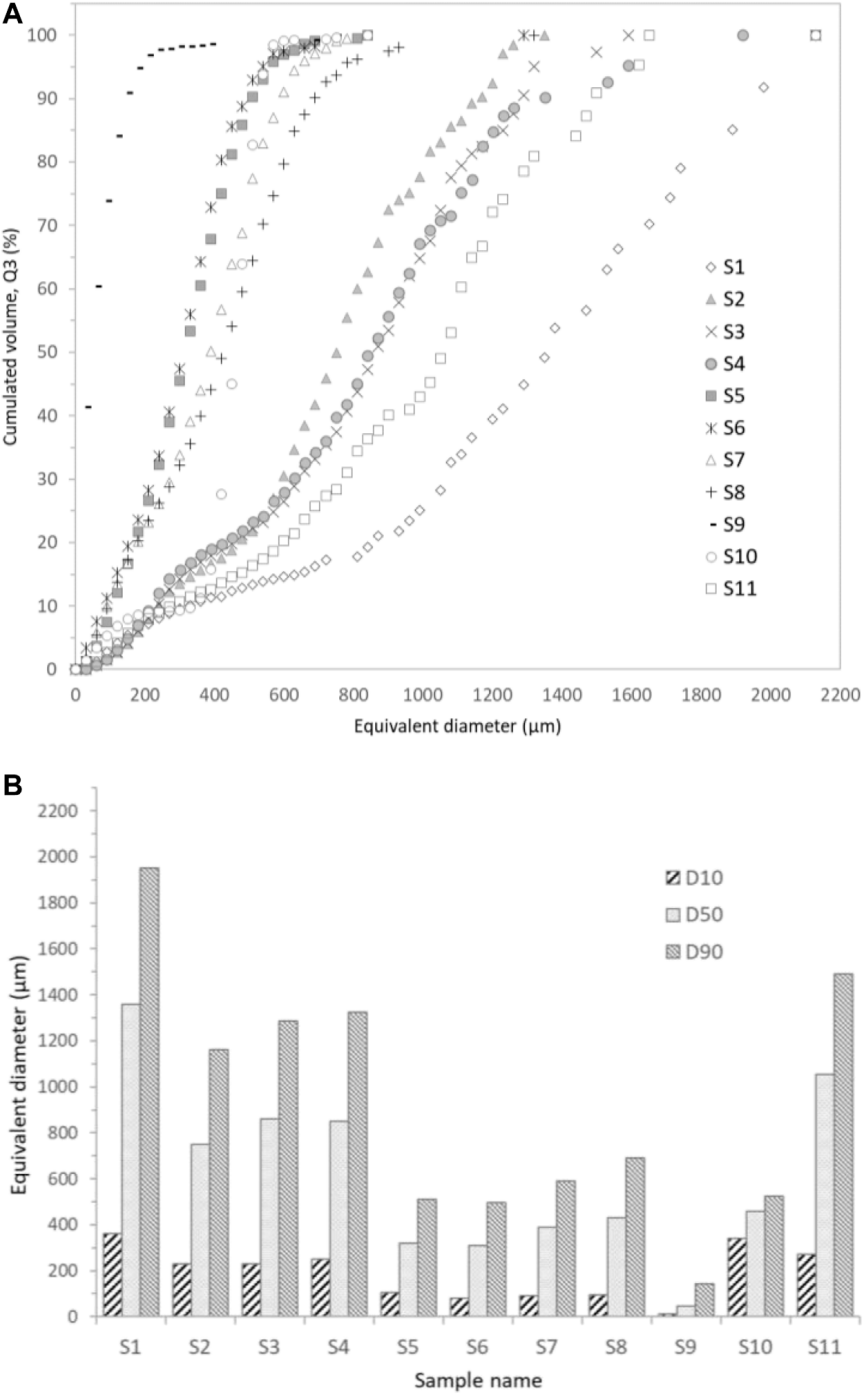
**(A)** Pore size distributions for tested samples. These distributions are expressed in percentage of cumulative volume (Q3). Each bin is 30 µm wide. **(B)** Pore size equivalent diameters D 10, D 50, and D 90 for all the tested samples.

Figures 8C,D present a 3D representation of the geopolymer with various vizualizations namely as solid surface and throats visualization or as pore segmentation (a) and (b) respectively or as connected or percolated pores respectively. The pore throats are colored according to their respective sizes, the pore cells are colored according to their label, and the percolated network is represented in pink color in the cross-section representation. As expected, the *Dn* values gathered in Table 5 are relatively large as compared to S3 and S7 samples, which were produced with lower H_2_O_2_ (but with the same surfactant). In general, S11 sample presents the higher mean equivalent pore and throat size due to the larger value of the hydrogen peroxide initially introduced in the paste (except for the S1 sample but due to the low volume of paste and H_2_O_2_, the uncertainties are quite large), Figures 8, 9. The analysis of the pore connectivity shows that the pore volume are fully percolated (Table 4).

## 4 Discussion

Foam microstructure is influenced by the nature of the surfactant added to the geopolymer paste to stabilize the gas bubbles and the quantity of blowing agent. Figure 9A proposes the cumulated volume (Q3) as a function of the equivalent diameter for all the analyzed samples. When the initial concentration of H_2_O_2_ (R = 0.25% for S9 and R = 0.5% corresponding to S5-S8 samples) is low, the equivalent diameter and its classes *Dn* (Figure 9B) are low. On the other hand, an increase of the gas source term produces a more aerated foam with bigger equivalent pore and throat diameters (S1-S4 and S11) except for the S10 sample that was synthetized with CTAB surfactant (but with R = 1%), which drastically modify the viscosity of the paste.

In the field of decontamination process of liquid wastes, we seek fully connected macroporous network that will facilitate the transport of fluid through the material. By this way, the total impregnation of the foam by the liquid waste to decontaminate will be promote. One key parameter to quantify the capacity of a material to ease the transport of fluid is the permeability that can be estimate from the Darcy equation (Eq. 1). Permeability may be predicted from tomography analysis (PNM) and specifically from the theoretical procedure explain in the section 2.3.4. According to the chemical formulation, the intrinsec permeability are ranged between 4.4 10^−17^ and 1.2 10^−10^ m^2^ (Table 5). For three samples, it was not possible to compute the permeability due to a non-percolated network (Table 5). As expected, the lower value of intrinsec permeability correspond to the foams that are synthetised by lower H_2_O_2_ and CTAB surfactant. This is due to the low value of throat size between two adjacent cells. For AER5 samples generated with R = 1 or 1.25%v/v, the permeability values are around 10^−11^ and 10^−10^ m^2^ that are values closed to those found for gravels or sands (Coussy, 2003). Another way to calculate the permeability consists in using a classical Kozeny-Carman (KC) equation in a laminar conditions (Schulz et al., 2019), such as:

$$\text{K}=\frac{{\text{D}}_{\text{p}}^{2}\epsilon^{3}}{180{\left(1-\text{ε}\right)}^{2}}$$ (2)

**FIGURE 10:**
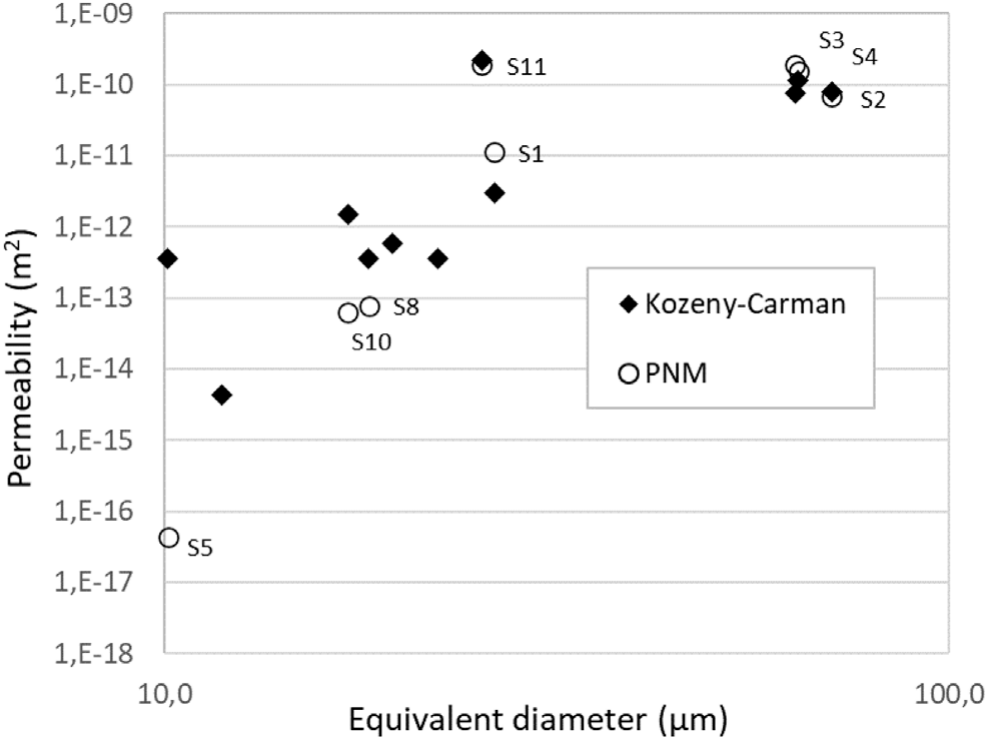
Plot of the permeability as a function of Throat diameters (inscribing diameters); inscribed disk diameter are used for Kozeny-Carman model and to describe the hydraulic diameter of throats for the PNM.

Where Dp is the pore diameters and ε is the total porosity. The D_10_ values of the pore throats, reported on Table 5, correspond to the limiting step for the transport of fluid. We use this Pore diameter definition to estimate the permeability form Eq. 2.180 is a parameter that depends on some geometrical considerations as tortuosity for example. The values obtained are of the same order of magnitude that those calculated from PNM as depicted in Figure 10. (Planel et al., 2020) calculated intrinsec permeability for geopolymer emulsion but computed the water permeability for both level of porous network: through the geopolymer mesoporous network, they find around 10^−18^ m^2^ and through the emulsion network where they find aound 10^−14^ m^2^. In our case, we added another level of macroporosity to reach a permeability around 10^−10^ m^2^. The fact that this level of permeability was reached allowed us to be confident for using these geopolymer foams as a filter in a continuous way because the linear pressure drop will be limited and the transport of fluid maximized especially by maximizing the porosity and the size of the pore throats.

All the results and the input data obtained from tomography are listed in Table 5; Figure 9. Different pores and throats size definitions could be used for the Kozeny-Carman model and for the PNM simulation that strongly impact the results. PNM simulation uses local hydraulic conductances between adjacent pores that requires the definition of hydraulic diameter of pores and throats joining these pores. Finally, inscribing disk diameter and the equivalent sphere diameter have been used for the definition of the hydraulic diameter of throats and pores respectively. Inscribing disk diameter of throats have been also chosen for the definition of the global pore size of the Kozeny-Carman model. Good correlations between PNM simulations and Kozeny-Carman model have been obtained with these pore/throats size definitions.

## 5 Conclusion

This paper investigated the characterization of macroporous network of a geopolymer foam and its properties as a function of the chemical formulation and its consequence in terms of transport of fluid through the computation of the permeability. X-ray tomography was found to be perfectly suitable for characterizing this kind of porous materials due to the probed length scale. This nondestructive method of characterization was then used to quantify the 3D morphology of pores such as porosity, cell and throat sizes, connectivity or anisotropy.

The nature of surfactant and the initial concentration of blowing agent H_2_O_2_ have a startling effect on the morphology and topology of the porous network. The CTAB based geopolymer consists in a more densely packed closed faceted pores, whereas the AER5 based foam consists in a 3D network of interconnected pores. The AER5-based geopolymer foam have a higher mean cell size that the cells of CTAB -based foams. On the other side, the increase of gas source term (higher initial concentration of H_2_O_2_) combined with AER5 surfactant produces more aerated foams with fully connected macroporous network. This monolith with open macroporosity and owing sufficient mechanical properties facilitates the transport of aqueous waste to decontaminate by limiting the pressure drop in the geopolymer filter. The permeability was assessed from tomography results and from the KC relationship for all synthetized geopolymer foams. A value of 10^−10^ m^2^ comparable to sand materials was reached for a chemical formulation that is considered as interesting for nuclear liquid waste decontamination.

## Data Availability

The datasets presented in this study can be found in online repositories. The names of the repository/repositories and accession number(s) can be found in the article/supplementary material.
